# De Novo RNA Sequencing and Expression Analysis of *Aconitum carmichaelii* to Analyze Key Genes Involved in the Biosynthesis of Diterpene Alkaloids

**DOI:** 10.3390/molecules22122155

**Published:** 2017-12-05

**Authors:** Megha Rai, Amit Rai, Noriaki Kawano, Kayo Yoshimatsu, Hiroki Takahashi, Hideyuki Suzuki, Nobuo Kawahara, Kazuki Saito, Mami Yamazaki

**Affiliations:** 1 Graduate School of Pharmaceutical Sciences, Chiba University, Chiba 260-8675, Japan; megha@chiba-u.jp (M.R.); ksaito@faculty.chiba-u.jp (K.S.); 2 Tsukuba Division, Research Center for Medicinal Plant Resources, National Institutes of Biomedical Innovation, Health and Nutrition, Tsukuba 305-0843, Japan; nkawano@nibiohn.go.jp (N.K.); yoshimat@nibiohn.go.jp (K.Y.); kawahara@nibiohn.go.jp (N.K.); 3 Medical Mycology Research Center, Chiba University, Chiba 260-8673, Japan; hiroki.takahashi@chiba-u.jp; 4 Kazusa DNA Research Institute, Chiba 292-0818, Japan; hsuzuki@kazusa.or.jp

**Keywords:** *Aconitum*, RNA sequencing, transcript abundance, diterpene alkaloids, aconitine, KEGG pathway enrichment analysis

## Abstract

*Aconitum carmichaelii* is an important medicinal herb used widely in China, Japan, India, Korea, and other Asian countries. While extensive research on the characterization of metabolic extracts of *A. carmichaelii* has shown accumulation of numerous bioactive metabolites including aconitine and aconitine-type diterpene alkaloids, its biosynthetic pathway remains largely unknown. Biosynthesis of these secondary metabolites is tightly controlled and mostly occurs in a tissue-specific manner; therefore, transcriptome analysis across multiple tissues is an attractive method to identify the molecular components involved for further functional characterization. In order to understand the biosynthesis of secondary metabolites, Illumina-based deep transcriptome profiling and analysis was performed for four tissues (flower, bud, leaf, and root) of *A. carmichaelii*, resulting in 5.5 Gbps clean RNA-seq reads assembled into 128,183 unigenes. Unigenes annotated as possible rate-determining steps of an aconitine-type biosynthetic pathway were highly expressed in the root, in accordance with previous reports describing the root as the accumulation site for these metabolites. We also identified 21 unigenes annotated as cytochrome P450s and highly expressed in roots, which represent candidate unigenes involved in the diversification of secondary metabolites. Comparative transcriptome analysis of *A. carmichaelii* with *A. heterophyllum* identified 20,232 orthogroups, representing 30,633 unigenes of *A. carmichaelii*, gene ontology enrichment analysis of which revealed essential biological process together with a secondary metabolic process to be highly enriched. Unigenes identified in this study are strong candidates for aconitine-type diterpene alkaloid biosynthesis, and will serve as useful resources for further validation studies.

## 1. Introduction

Plant species from the *Aconitum* genus are described as one of “The Four Pillars” of the ancient herbs used in Chinese medical healing. Approximately 76 species of *Aconitum* genus are used for medicinal purposes and are called ‘Aconites’, with *Aconitum carmichaelii* being the most widely used herb [1,2]. *A. carmichaelii*, native to eastern parts of Asia and Russia, is a perennial plant growing up to a height of 60–150 cm with dark brown roots, palmately divided dark-green glossy leaves, and violet flowers with helmet-shaped petals [3]. Principal and lateral roots of *A. carmichaelii* are used as medicine under the names “Chuanwu” and “Fuzi” (also called “Bushi” in Japan), respectively, with Fuzi being preferred in most traditional medicine formulations [2,4]. Root extract of *A. carmichaelii* is used for the treatment of several medical conditions including collapse, rheumatic fever, painful joints, gastroenteritis, diarrhea, bronchial asthma, and edema [2,3,5,6]. Kampo medicine formulations employing “Bushi” have also been reported to be effective for several types of neuropathic pain [7,8,9].

The pharmacological properties of *A. carmichaelii* are attributed to the accumulation of diverse range of bioactive C_20_-, C_19_- and C_18_-type diterpene alkaloids, which can be broadly classified into three types, namely, diester–diterpene alkaloids, monoester–diterpene alkaloids and unesterified diterpene alkaloids. The majority of the C_19_-type diterpene alkaloid skeletons, which represent the major alkaloid content of *A. carmichaelii*, are derived from various substitutions of the aconitine skeleton, leading to a total of 76 different alkaloids [6]. 

Biosynthesis of diterpene alkaloids is initiated with the condensation of three molecules of isopentenyl pyrophosphate (IPP) into geranylgeranyl pyrophosphate (GGPP) in the presence of enzyme geranylgeranyl pyrophosphate synthase (GGPPS) [10,11]. GGPP thus formed undergoes proton-induced cyclization by copalyl–diphosphate synthase (CDPS) to form copalyl diphosphate. Copalyl-diphosphate either undergoes further cyclization and rearrangement by kaurene synthase (KS) to form kaurene or through alternate rearrangement reaction forms atisane. Further, atisane and kaurene are oxidized and hydroxylated in the subsequent steps, and by the incorporation of β-ethanolmaine, which is derived from decarboxylation of l-Serine, forms an atisine skeleton. The atisine skeleton can interconvert to a veatchine skeleton, and further oxidation and modification of the scaffold skeletal mediated by cytochrome P450s may occur, leading to the formation of different C_20_-type diterpene alkaloid skeletons. C_20_-type diterpene alkaloids thus formed may undergo demethylation, which eventually gives rise to the C_19_-type and C_18_-type diterpene alkaloid skeleton [12,13]. 

While numerous studies have reported the levels and types of diterpene alkaloids in different tissues of *A. carmichaelii* [14,15,16,17], the genome and transcriptome data are limited, with only 84 sequences being reported to date in the NCBI database for *A. carmichaelii*, 56 of which are associated with chloroplasts. The lack of genomic and transcriptomic resources serves as a major roadblock in the identification of molecular components contributing to the biosynthesis of specialized metabolites of *A. carmichaelii*. Pal et al. recently described de novo transcriptome assembly characteristics of *A. heterophyllum*, a non-toxic plant species from *Aconitum* genus using root and shoot tissues. *A. heterophyllum* does not produce toxic diester–diterpene alkaloids, with atisine being the major alkaloid [18,19]. While de novo transcriptome assembly and expression analysis of *A. heterophyllum* are important resources, they are of limited use when studying the biosynthesis of highly toxic aconitine-type diterpene alkaloids, which are the signature metabolites for the majority of aconites. Therefore, de novo transcriptome assembly and characterization of toxic aconites, together with differential expression analysis of tissues with different accumulation patterns of aconitine-type diterpene alkaloids, is highly desired to understand their biosynthesis and regulation. Furthermore, comparative transcriptome analysis of aconitine producing aconite and *A. heterophyllum* will be useful to identify potential candidate genes involved in the biosynthesis of aconitine-type diterpene alkaloids, and may provide important clues on the evolution of *A. heterophyllum* as a relatively less toxic aconite. 

In this study, we established de novo transcriptome assembly of *A. carmichaelii* using four tissue types, namely flower, bud, leaf, and root. Through sequence-homology based transcriptome annotation coupled with co-expression analysis, we identified candidate unigenes involved in the biosynthesis of aconitine-type diterpene alkaloids. This study, representing the first transcriptomic resource of *A. carmichaelii*, lays a foundation for future studies on the functional characterization of candidate genes involved in the biosynthesis of pharmacologically active diterpene alkaloids, which could help to develop a genetic intervention strategy for enhancing the production of pharmacologically important metabolites. 

## 2. Results and Discussion

### 2.1. Sample Preparation and High-Throughput Transcriptome Sequencing

**Figure 1:**
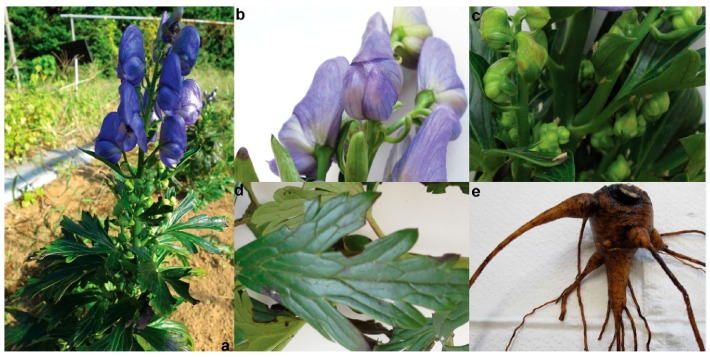
Tissues of *A. carmichaelii* used for de novo transcriptome assembly. (**a**) *A. carmichaelii* plant growing in natural condition; (**b**) flower; (**c**) bud; (**d**) leaf; and (**e**) root.

Since no genomic and transcriptomic resources for *A. carmichaelii* were available, we performed RNA-seq based deep transcriptome profiling to derive de novo transcriptome assembly, which was further used for the comparative transcriptome analysis with *A. heterophyllum* and transcript expression analysis across four tissues of *A. carmichaelii*. In order to achieve complete representation of *A. carmichaelii* transcriptome, total RNA from four tissues of *A. carmichaelii*, namely, flower, bud, leaf, and root, was extracted (Figure 1). RNA samples with an RNA Integrity Number value over 8 were used for mRNA preparation, fragmentation, cDNA synthesis, and library preparation. Individual libraries for each tissue thus prepared were sequenced using the Illumina HiSeq™ 2000 platform in the paired-end mode, resulting in a total of about 6.1 Gbps sequence reads, with a mean length of raw read of 101 bps. Raw reads, after being processed to remove adaptor sequences, low-quality reads (Phred score < 30), and reads shorter than 50 bps, resulted in about 5.5 Gbps sequencing datasets, which were used for de novo transcriptome assembly. The average Phred score, which is a measure of the quality of the sequence reads [20], for each library was above 36 and trimmomatic program-based pre-processing dropped <1% of the total sequence data (Table S1), suggesting that sequence reads generated in this study are of high quality for its subsequent use in generating de novo transcriptome assembly.

### 2.2. De Novo Transcriptome Assembly

The first step towards transcriptome analysis of a plant species without a reference genome is to obtain a high-quality de novo transcriptome assembly. Advancements in next-generation sequencing technologies have led to the development of several computational tools and algorithms to assemble the generated sequence datasets de novo [21]. Numerous studies have shown that there is no single assembler that works best for all plant species, and therefore it is important to test multiple assemblers with varying parameters to obtain the most suitable transcriptome assembly for subsequent analysis [22,23]. Here, we tested three popular de novo transcriptome assemblers, namely CLC Genomics Workbench 8.0.3 (https://www.qiagenbioinformatics.com/), SOAPdenovo-Trans [24], and Trinity [25], and the assembly statistics for resulting de novo transcriptome assemblies are summarized in Table S2. Using default assembly parameters, CLC Genomics Workbench assembled 44.1 Mbps into 88,873 contigs with an N50 value of 554 bps. Using SOAPdenovo-Trans, we generated de novo transcriptome assembly using k-mer sizes of 31, 41, 51, 63, 71 and 91, resulting in six different assemblies. SOAPdenovo-Trans with k-mer size of 31 resulted in the best assembly statistics with 39.53 Mbps assembled into 268,504 contigs and an N50 value of 682 bps. De novo transcriptome assembly using Trinity program with its default parameters assembled 117.4 Mbps into 182,928 transcripts, corresponding to 114,268 trinity-genes with a mean length of 642.3 bps and N50 value of 905 bps. The transcriptome assembly derived from the Trinity program resulted in better assembly statistics with longer sequences and higher N50 value when compared with assemblies obtained from other programs and hence was selected for subsequent analysis. The Trinity-derived de novo transcriptome assembly was further processed through CD-HIT-EST program [26] to remove transcript redundancies, resulting in a final assembly of 128,183 unigenes with an N50 value of 830 bps (Table 1). The length distribution of our assembled transcripts revealed that 46,697 and 19,913 unigenes were longer than 500 and 1000 bps, respectively (Figure S1). The transcriptome assembly thus obtained was used for further annotation and characterization.

### 2.3. Functional Annotation of Unigenes

**Figure 2:**
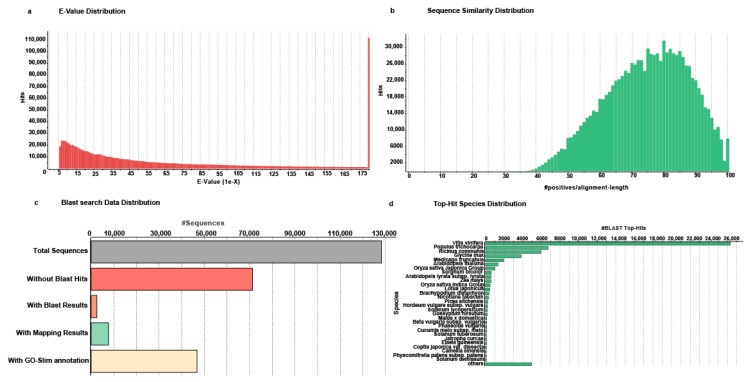
Characterization of *A. carmichaelii* unigenes using Blastx program against the NCBI-non-redundant (nr) database. (**a**) *E*-value distribution of top Blastx hits for each unigene; (**b**) sequence similarity distribution plot based on the top Blastx hits for the annotated unigenes; (**c**) Blastx search-based data distribution; (**d**) species distribution of the top Blastx hits of annotated unigenes.

Sequence-homology-based annotation is the first step towards assigning putative functions to the unigenes; it serves as the basis of hypothesis generation and further validation, and offers insight into different ongoing biological processes within a system [27,28]. In order to annotate and characterize de novo transcriptome assembly of *A. carmichaelii*, we performed a Blastx [29] sequence homology search against the NCBI non-redundant (nr) protein database with *E*-value cutoff < 10^−5^ and maximum number of allowed hits for each query set a 20, and the top Blastx hits were selected to annotate the corresponding query unigenes. Blastx search results showed that over 85% of the aligned unigenes had significant homology with the sequences in the NCBI-nr database (Figure 2a), with over 26,949 unigenes exhibiting sequence similarity with corresponding hits for over 80% (Figure 2b). Blastx-based annotation of unigenes is summarized in Table S3. Blastx search results were further analyzed using Blast2GO program v 4.0 [30] to obtain gene ontology (GO) terms, to extract enzyme commission (EC) number, and to associate Kyoto Encyclopedia of Genes and Genomes (KEGG) pathway-based information with the assembled unigenes of *A. carmichaelii* transcriptome assembly. Briefly, of the 128,183 unigenes, 56,928 unigenes got a blastx search hit in the database and 46,742 unigenes were assigned a plant GO-slim-based annotation using the Blast2GO program (Figure 2c). Top-hit species distribution analysis of *A. carmichaelii* transcriptome assembly showed that 80% of its annotated unigenes had a high sequence similarity with the homologs of *Vitis vinifera*, *Populous trichocarpa*, *Ricinus communis*, *Medicago trunctula*, and *Arabidopsis thaliana* (Figure 2d).

**Figure 3:**
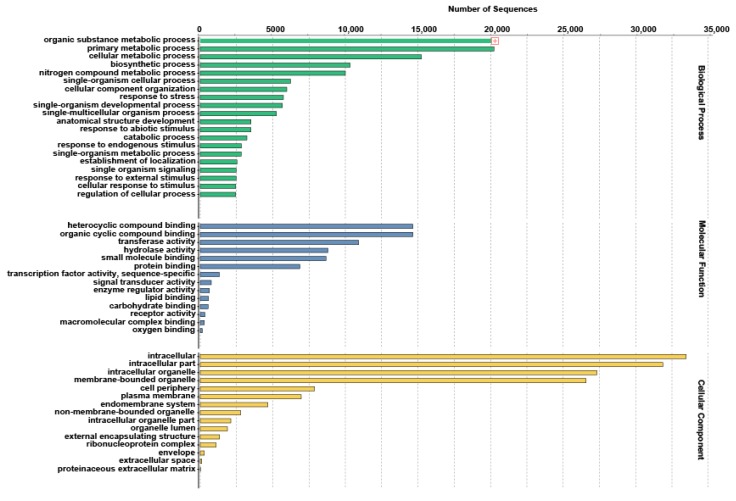
Gene ontology (GO) distribution in de novo transcriptome assembly of *A. carmichaelii*. GO terms for all annotated unigenes based on Blastx results at GO annotation level 3 are summarized in three functional categories: biological processes (BP), molecular function (MF), and cellular component (CC).

GO-based functional classification of *A. carmichaelii* transcriptome assembly at level 3 assigned all annotated unigenes into 98 different GO terms in three broad categories: biological process, molecular Function, and cellular Component. Within the biological process GO category, the top five categories in terms of number of unigenes assigned included organic substance metabolic process, primary metabolic process, cellular metabolic process, biosynthetic process, and nitrogen compound metabolic process. In the molecular function GO category, GO terms corresponding to heterocyclic compound binding, organic cyclic compound binding, transferase activity, hydrolase activity, and small molecule binding were the most abundant among the assigned unigenes. For the cellular component GO category, the majority of unigenes were classified in GO terms corresponding to intracellular, intracellular part, intracellular organelle, membrane-bound organelle, and cell periphery (Figure 3).

### 2.4. KEGG Database-Based Functional Characterization of A. carmichaelii Transcriptome Assembly

**Figure 4:**
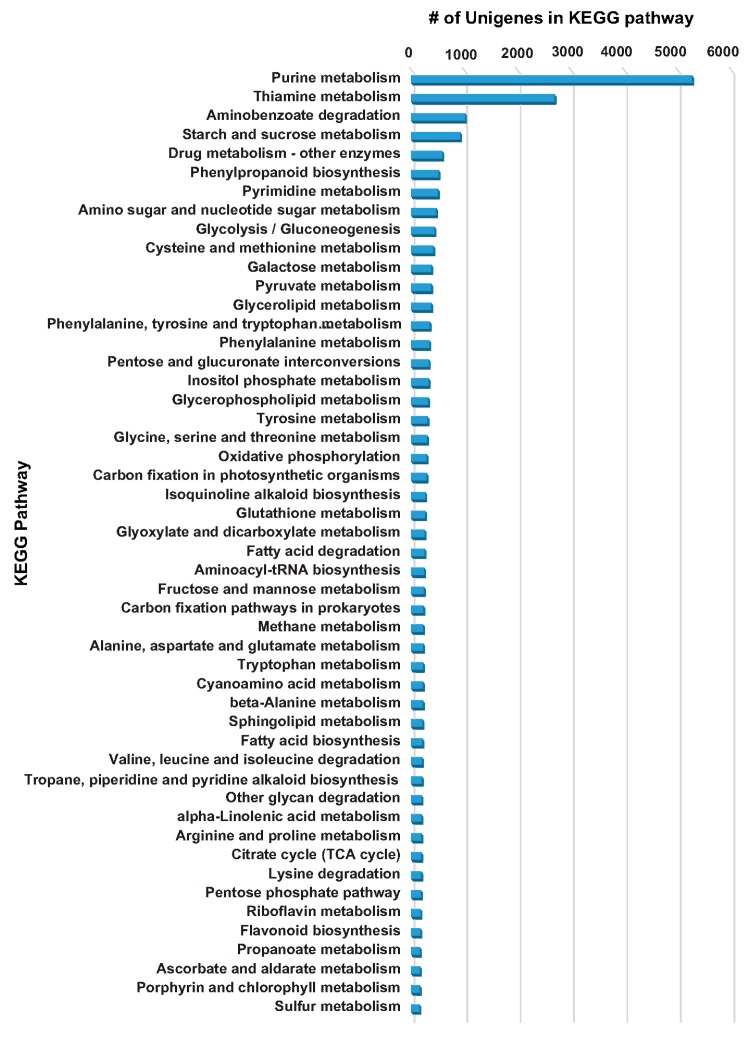
Functional annotation of the *A. carmichaelii* transcriptome assembly by KEGG pathways database. Top 50 KEGG pathways based on the number of assigned unigenes of *A. carmichaelii* transcriptome assembly.

The KEGG pathway database provides systematic knowledge of genes’ functions and their interactions/relationships with other cellular components, and links genomic information with high-order functional information [31,32]. Such “pathway”-based unigene mapping and classification provide a broad overview of different ongoing biological and metabolic processes within an organism. The pathway-based annotation coupled with transcript expression analysis and knowledge of key metabolite accumulation trends across different tissues also give an important insight into the biosynthesis and regulation of metabolic pathway of interest. KEGG pathway mapping of *A. carmichaelii* de novo transcriptome assembly based on Blastx annotation was performed using Blast2GO software v 4.0, resulting in 10,606 unigenes being assigned to 131 KEGG pathways. The top 50 KEGG pathways on the basis of number of unigenes assigned are shown in Figure 4. Purine metabolism, thiamine metabolism, aminobenzoate degradation, starch and sucrose metabolism, and drug metabolism (other enzymes) were the top five KEGG pathways assigned to *A. carmichaelii* transcriptome assembly. Among the key KEGG pathways listed in the top 50 were glycine, serine, and threonine metabolism; isoquinoline alkaloid biosynthesis; glutathione metabolism; and tropane, piperidine, and pyridine alkaloid biosynthesis. Metabolic pathways such as terpenoid backbone synthesis and diterpenoid biosynthesis were assigned to 149 and 78 unigenes, respectively (Table S4). KEGG database-based functional characterization of *A. carmichaelii* transcriptome assembly showed unigenes assigned to all major specialized biosynthetic pathways that are known or expected to be present in *A. carmichaelii*.

### 2.5. Identification of Simple Sequence Repeats (SSRs)

**Figure 5:**
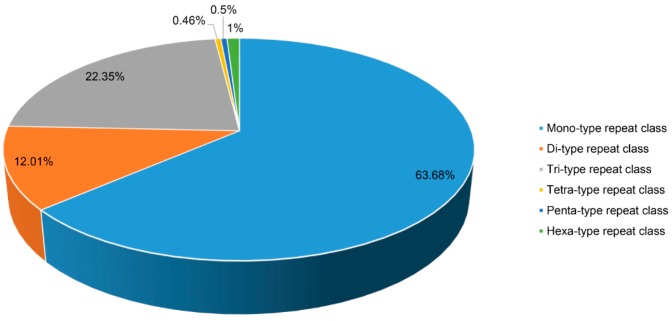
SSRs detected in *A. carmichaelii* transcriptome assembly. Distribution of different repeat type classes of SSR.

SSRs are mutation-prone DNA regions ubiquitously distributed across the genome, containing tandem repetitions (typically 5–50 times) of certain simple motifs of 1–6 nucleotides at a locus. SSRs are widely used as molecular markers due to several advantages including high abundance, uniform distribution, and hypervariability, among others [33]. SSRs serves as an important resource to access genetic diversity, to determine genetic variations including paternity determination, for population genetic studies, and for the development of genetic maps [34,35]. In order to identify SSRs of *A. carmichaelii*, the de novo transcriptome assembly was screened to identify mono- to hexa-nucleotide motifs with at least 10 repetitions using MISA software [36], resulting in a total of 16,068 SSR across 14,168 unigenes, with 1675 unigenes containing more than one SSR (Table 2). Within identified SSRs in *A. carmichaelii* transcriptome assembly, mono-nucleotide repeats had the highest frequency (63.68%) of all SSRs, followed by tri- and di-nucleotide repeats (22.3% and 12%, respectively), while other repeat-type class frequencies were relatively small (Figure 5). All the identified SSRs of *A. carmichaelii* have been listed in Table S5.

Since the scope of our study also included an overall comparison between the transcriptome assemblies of *A. carmichaelii* and *A. heterophyllum*, the individual de novo transcriptome assemblies of root and shoot tissue of *A. heterophyllum* were downloaded (http://14.139.240.55/NGS/download.php) and a de novo transcriptome assembly was derived by combining the published assemblies of root and shoot tissues of *A. heterophyllum* and processing them through the CD-HIT-EST program to remove redundancies. De novo transcriptome assemblies were re-annotated using the Blastx program against the NCBI-nr protein database. The de novo transcriptome assembly of *A. heterophyllum* was used for identification of SSRs, as described for *A. carmichaelii*. SSRs analysis revealed that, similar to *A. carmichaelii*, mono-nucleotide (61%) repeat was represented by the largest fraction of all SSRs in *A. heterophyllum*, followed by tri- (25.37%) and di-nucleotide (12.42%) repeats (Tables S6 and S7). The SSRs of *A. carmichaelii* and *A. hetreophyllum* identified herein provide a potential genetic marker, and are an important resource for genetic diversity assessment and comparative genetics across different *Aconitum* species.

### 2.6. Functional Classification of Tissue-Specific Unigenes of A. carmichaelii

**Figure 6:**
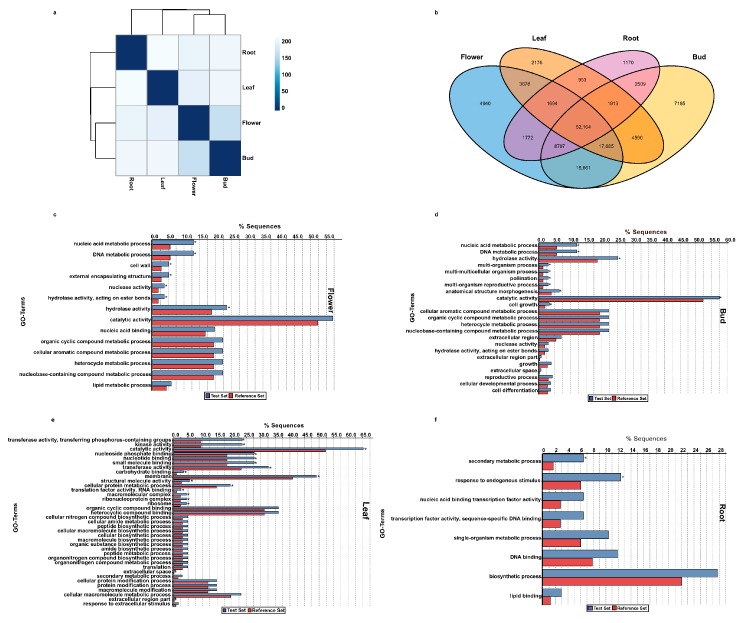
Transcriptome expression analysis across four tissues. (**a**) Euclidean distance-based hierarchical clustering using expression value of unigenes with non-zero FPKM across four tissues of *A. carmichaelii*; (**b**) Venn diagram of tissue-specific unigenes (FPKM > 0); (**c**–**f**) GO enrichment analysis for tissue-specific unigenes. Using tissue-specific unigenes as a test set and unigenes of *A. carmichaelii* with GO annotation as a reference set, gene ontology enrichment analysis was performed using Fisher’s exact test with the *p* value cut-off set as < 0.05. * represents enriched GO terms with FDR < 0.05.

Plant tissues are specialized to perform specific functions, and are the sites for the accumulation of several important secondary metabolites [37]. Transcript expression analysis across different tissues provides important clues about the different ongoing cellular and metabolic processes that are common across all tissues or are unique to a specific tissue type. In order to gain insight into the different shared/unique biological and metabolic processes across four tissues of *A. carmichaelii* at transcript expression level, clean paired-end reads were aligned to the de novo transcriptome assembly using the Bowtie 2 program [38], and normalized transcript expression in FPKM (Fragments Per Kilobase of transcript per Million mapped reads) were determined by the RSEM program [39]. Euclidean distance-based hierarchical clustering using expression of unigenes across four tissues of *A. carmichaelii* showed tissues being clustered in three groups (Figure 6a). While flower and bud tissues were grouped in one cluster, leaf and root formed a separate cluster, suggesting a similar transcriptome expression profile within flower and bud, and a distinct expression profile between members of individual clusters. Flower and bud tissues share numerous properties including function, features, and type of metabolites being synthesized to a large extent, and so the similarity in terms of overall expression profile between them was expected. Our results thus suggest that the transcriptome profiling data of the four tissues of *A. carmichaelii* effectively captured the distinct biological processes of the four tissues.

Transcriptome expression analysis for the four tissues of *A. carmichaelii* showed 106,391, 110,504, 84,853, and 70,972 unigenes with active expression (non-zero FPKM values) in the flower, bud, leaf, and root, respectively. Among actively expressed unigenes, 52,164 were common across all the four tissues of *A. carmichaelii*, while 4940, 7185, 2176 and 1170 unigenes were specifically expressed in the flower, bud, leaf, and root, respectively (Figure 6b and Table S8). Flower, bud, and leaf tissues of *A. carmichaelii* shared 17,685 unigenes with non-zero FPKM values, while only 1913 unigenes were shared between bud, leaf, and root. Our results showed that, in general, flower and bud tissues of *A. carmichaelii* shared more transcriptionally active unigenes with other tissues, while root and leaf showed a distinct set of transcriptionally active unigenes.

In order to further understand the functional classification of tissue-specific transcriptionally active unigenes, we performed GO enrichment analysis using Fisher’s exact test with tissue-specific unigenes as the test set and unigenes of *A. carmichaelii* with GO annotation as the reference set. Noticeably, among the major GO terms enriched in the flower-specific transcriptionally active unigenes were GO terms corresponding to the organic cyclic compound metabolic process and the cellular aromatic compound metabolic process, while GO terms corresponding to pollination, anatomical structure morphogenesis, the cellular aromatic compound metabolic process, and the organic cyclic compound metabolic process were highly enriched in the bud-specific transcriptionally active unigenes (Figure 6c,d). The aromatic compound biosynthetic process and the pollination process being enriched in flower-specific and bud-specific unigenes is consistent with the specialized function of these tissues, and hence further validates our approach to the annotation and characterization of *A. carmichaelii* transcriptome assembly. GO terms enriched in the leaf-specific transcriptionally active unigenes included processes such as transferase activity, transferring phosphorus-containing groups, kinase activity, catalytic activity, and nucleoside phosphate binding (Figure 6e). It is interesting to note that the root, which is the primary site of accumulation of major diterpene alkaloids of *A. carmichaelii*, showed GO terms corresponding to secondary metabolic process being significantly enriched (Figure 6f). Furthermore, tissue-specific transcriptionally active unigenes for the flower, bud, leaf and root of *A. carmichaelii* showed 135, 204, 107 and 33 of the unigenes being mapped to different KEGG pathways, respectively. KEGG pathway enrichment analysis of the tissue-specific unigenes revealed that the amino sugar and nucleotide sugar metabolism pathway (map00520) was significantly enriched in the flower, bud, and leaf, while galactose metabolism (map00052), thiamine metabolism, (map00730) and oxidative phosphorylation (map00190) were significantly enriched in the flower and bud of *A. carmichaelii*. The aminobenzoate degradation pathway (map00627) was significantly enriched in the flower and leaf, while glyoxylate and dicarboxylate metabolism (map00630) and purine metabolism (map00230) were enriched in the bud and leaf. Interestingly, monoterpenoid biosynthesis (map00902), which plays a significant role in flower fragrance, was enriched in flowers, while diterpenoid biosynthesis (map00904) was significantly enriched in roots of *A. carmichaleii* in accordance with the accumulation pattern of diterpene alkaloids in *A. carmichaelii*, thus validating our approach to KEGG enrichment analysis (Table S9). The root-specific unigenes assigned to the diterpenoid biosynthesis pathway included TR60126|co_g1_i1 and TR48303|c1_g4_i1, both of which were annotated as CDPS, the first enzyme driving GGPP towards the biosynthesis of diterpene alkaloids. Taken together, our results suggest that the transcriptome dataset for four tissues of *A. carmichaelii* has captured relevant biological processes.

### 2.7. Identification of Orthologous Unigenes and Transcriptome Divergence between A. carmichaelii and A. heterophyllum

Comparative transcriptome analysis using de novo transcriptome assemblies of plant species from the same or distant families offers important insights into the molecular components participating in the biosynthesis of species-specific or shared specialized metabolites [40]. Such analysis also assists in the identification of orthologous genes and reduces the number of false positives for further differential expression analysis. While our study is the first report to date on the de novo transcriptome assembly and expression analysis of aconitine-producing aconites, a transcriptome assembly of the non-aconitine-producing plant from the *Aconitum* genus, *A. heterophyllum*, offers us a unique resource [18]. Comparing the de novo transcriptome assembly of *A. carmichaelli* obtained in this study with that of *A. heterophyllum* would allow us to identify transcripts that are shared between these two species or are unique to *A. carmichaelii*, which will be important in identifying strong candidate genes associated with aconitine biosynthesis. Orthologous and species-specific unigenes were identified using the OrthoFinder program, which solves the problem of gene length bias in blast searches and outperforms the commonly used OrthoMCL in speed and accuracy [41]. De novo transcriptome assemblies of *A. heterophyllum* and *A. carmichaelii* were translated as described in the material and method Section 3.8, resulting in 128,183 and 81,607 nigenes, respectively, being translated and used as input for OrthoFinder analysis. OrthoFinder analysis resulted in a total of 20,247 orthogroups consisting of 30,633 and 26,282 unigenes of *A. carmichaelii* and *A. heterophyllum*, respectively. Among orthogroups, while 20,232 orthogroups contained at least one unigenes from both of the species, seven and eight orthogroups were identified as species-specific orthogroups consisting of 48 and 39 unigenes of *A. carmichaelii* and *A. heterophyllum*, respectively (Table 3).

Overall, 72.9% of transcriptome datasets used for OrthoFinder analysis were not assigned to any orthogroups, and represented unique unigenes present in *A. carmichaelii* and *A. heterophyllum*. Additonal data for all the identified orthogroups and unique sequences are provided in Table S10.

**Figure 7:**
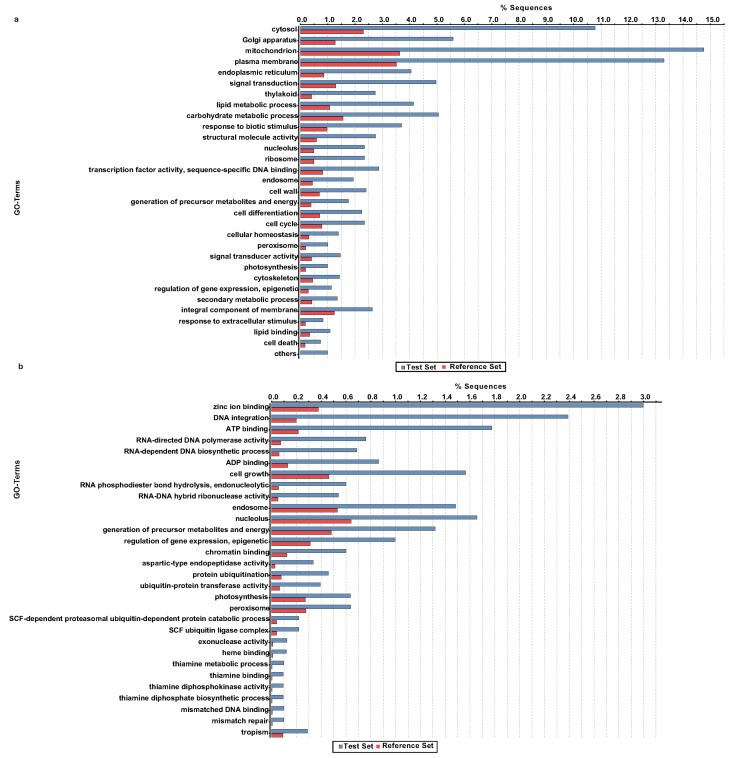
Comparative transcriptome analysis of *A. carmichaelii* and *A. heterophyllum*. (**a**) Gene ontology enrichment analysis for common orthologue unigenes between *A. carmichaelii* and *A. heterophyllum*; (**b**) gene ontology enrichment analysis for unassigned transcripts of *A. carmichaelii*. Using orthologue unigenes or unigenes unassigned to any clusters as a test set, respectively, and *A. carmichaelii* as the reference set, gene ontology enrichment analysis was performed using Fisher’s exact test with the *p* value cut-off set at <0.05.

In order to understand the functional classification of the orthologous unigenes between these two species, we performed GO enrichment analysis using Fisher’s exact test with unigenes of *A. carmichaelii* that had orthologous genes in *A. heterophyllum* as the test set and the *A. carmichaelli* transcriptome assembly as the reference set. The majority of the enriched GO terms were associated with essential biological processes including primary metabolism, cellular structure and components, cell cycle, signal transduction, and response to stimulus among several others (Figure 7a). It is noteworthy that the GO terms corresponding to the secondary metabolic process and transcription factor activity were also significantly enriched among the orthologous unigenes, which was expected since both plant species produce pharmacologically active diterpene alkaloids. Numerous studies in the past have identified and characterized transcription factors involved in the biosynthesis and regulation of diterpene alkaloids [42,43] and, therefore, shared unigenes noted as transcription factors in *A. carmichaelii* and *A. heterophyllum* are potential candidate regulators for further functional characterization. Among the unigenes of *A. carmichaelii* that were not assigned to any of the orthogroups, the GO enrichment analysis identified GO terms corresponding to zinc-ion binding, DNA integration, ATP binding, RNA-directed DNA polymerase activity, and RNA-dependent DNA biosynthetic process as the top five GO terms being enriched (Figure 7b).

### 2.8. Identification of Putative Candidate Unigenes Associated with the Biosynthesis of Aconitine and Its Derivatives

**Figure 8:**
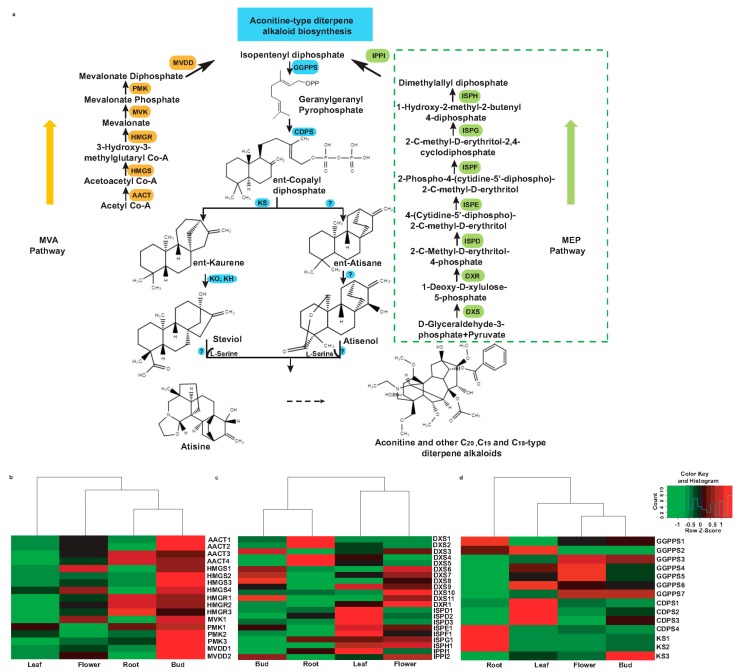
Schematics of the putative aconitine biosynthetic pathway, and the differential expression of the unigenes involved in *A. carmichaelii*. (**a**) Proposed pathway of aconitine biosynthesis; (**b**) expression of unigenes involved in MVA pathway; (**c**) expression of unigenes involved in MEP pathway; (**d**) expression of unigenes involved in diterpene alkaloid biosynthesis. Abbreviations: AACT (acetoacetyl-CoA thiolase), HMGR (3-hydroxy-3-methylglutaryl-CoA reductase), HMGS (3-hydroxy-3-methylglutaryl-CoA synthase), MVK (mevalonate kinase), PMK (phosphomevalonate kinase), MVDD (mevalonate diphosphate decarboxylase), DXS (1-deoxy-d-xylulose 5-phosphate synthase), DXR (1-deoxy-d-xylulose 5-phosphate reductoisomerase), ISPD (2-*C*-methyl-d-erythritol 4-phosphate cytidylyltransferase), ISPE (4-(cytidine-5′-diphospho)-2-*C*-methyl-d-erythritol kinase), ISPF (2-*C*-methyl-d-erythritol 2,4-cyclodiphosphate synthase), ISPG ((*E*)-4-hydroxy-3-methylbut-2-enyl diphosphate synthase), ISPH ((*E*)-4-hydroxy-3-methylbut-2-enyl diphosphate reductase), IPPI (isopentenyl diphosphate isomerase), GGPPS (geranylgeranyl pyrophosphate synthase), CDPS (ent-copalyl diphosphate synthase), KS (kaurene synthase), KO (Kaurene oxidase), and KH (Kaurenoic acid hydroxylase).

Aconitine and its derivatives such as mesaconitine, jesaconitine, and hypaconitine are key bioactive metabolites that convey medicinal properties to *A. carmichaelii* and other aconites, the complete biosynthetic pathway of which is largely unknown. A schematic diagram of the proposed pathway for the biosynthesis of aconitine and its derivatives is shown in Figure 8a. Precursors for the biosynthesis of aconitine-type diterpene alkaloids are derived from IPP, which is the end product of both the mevalonate (MVA) and methylerythritol (MEP) biosynthetic pathway. While classical biochemists describe the MEP pathway as the only contributor of IPP for biosynthesis of diterpene alkaloids, numerous studies have now established that the MVA pathway may also contribute to the biosynthesis of diterpene alkaloids in plants through the exchange of intermediates with the MEP pathway [18]. Among the earliest biochemical reactions towards the biosynthesis of diterpene alkaloids, three molecules of IPP condense to form GGPP in the presence of enzyme GGPPS [10,11]. GGPP then undergoes proton-induced cyclization and rearrangement to form kaurene or atisane. Furthermore, atisane and kaurene are oxidized and hydroxylated in the subsequent steps, and by the incorporation of β-ethanolmaine form an atisine skeleton [12,13]. Kaurene, which also serves as an intermediate gibberellin biosynthesis, is an important metabolic intermediate for the biosynthesis of diterpene alkaloids [44].

In order to identify candidate unigenes associated with aconitine biosynthesis, we screened the annotated transcriptome assembly of *A. carmichaelii* for the homologs corresponding to the 17 enzymes of the proposed biosynthetic pathway (Figure 8a), and used stringent criteria for selecting unigenes with length and sequence similarity with top Blastx hit over 500 bps and 70%, respectively, to retain the strongest candidates for the subsequent analysis. This resulted in the selection of a total of 52 unigenes associated with the MVA pathway (21 unigenes), MEP pathway (17 unigenes), and aconitine-type diterpene alkaloid biosynthetic pathway (14 unigenes), which is summarized in Table 4. Unigenes associated with the MVA pathway were highly expressed in the bud and root of *A. carmichaelii*, while moderate and low expression, respectively, were observed for the flower and leaf (Figure 8b). The MVA pathway contributes metabolic intermediates for the biosynthesis of sesquiterpenoids including aromatic compounds and plays an important role in attracting pollinators and seed disseminators [45,46]. Unigenes from the MVA pathway are highly expressed in the bud and flower, with enriched GO terms corresponding to the aromatic compound metabolic process being enriched in the bud-specific and flower-specific unigenes test set, which corroborates the biological functions of these tissues and further validates the quality of our transcriptome datasets. Unigenes associated with the MVA pathway showed a mixed expression trend for the roots of *A. carmichaelii*. While unigenes corresponding to HMGR (HMGR1, HMGR2, and HMGR3) and PMK (PMK1) were highly expressed in the root, the expression of unigenes corresponding to HMGS (HMGS1, HMGS2, HMGS3, HMGS4) MVK (MVK1), and MVDD (MVDD1, MVDD2) were relatively low compared to other tissues.

Homologs of all enzyme-coding genes of the MEP pathway were highly expressed in the leaf, while all but ISPD (ISPD1, ISPD2, and ISPD3) and ISPF (ISPF1) showed high expression in the flower of *A. carmichaelii* (Figure 8c). Buds showed high expression of unigenes, corresponding to DXS (DXS3, DXS6, DXS7, DXS8, and DXS11) and IPPI (IPPI2). Compared to these three tissues of *A. carmichaelii*, the root showed high expression for unigenes annotated as homologs of DXS (DXS1. DXS2, DXS4, and DXS5), and moderate expression for unigenes annotated as ISPG (ISPG1) and IPPI (IPPI2). High expression of unigenes corresponding to the homologs of plastidial MEP pathway in the leaf compared to the rest of the tissues was expected and further validates our de novo transcriptome assembly, annotation, filtering criteria, and expression analysis strategy.

In order to understand the biosynthetic pathway of aconitine biosynthesis post-IPP synthesis, we investigated the expression of three known genes associated with the biogenesis of aconitine-type diterpene alkaloids, namely, GGPPS, CDPS, and KS. Homologs of GGPPS (GGPPS1), CDPS (CDPS4), and KS (KS1, KS2) showed high expression in root of *A. carmichaelii* (Figure 8d). While homologs of one of the three genes associated with aconitine biosynthesis, GGPPS (GGPPS3, GGPPS4, GGPPS5, and GGPPS7), were highly expressed in flowers, homologs of both GGPPS (GGPPS7) and KS (KS3) were highly expressed in buds. For the leaf tissue of *A. carmichaelii*, unigenes annotated as GGPPS (GGPPS2 and GGPPS6) and CDPS (CDPS1, CDPS2, and CDPS3) were highly expressed, while homologs for KS showed low expression compared to the other three tissues. 

The expression values of all 52 unigenes associated with aconitine-type diterpene alkaloids (Table S11) were used to perform correlation analysis across four tissues of *A. carmichaelii* using Pearson’s distance matrix. Correlation analysis showed all of the 52 unigenes grouped into three major clusters, none of which included unigenes associated with the complete MEP/MVA pathway together with GGPPS, CDPS, and KS (Figure S2). Unigenes grouped in Cluster 1 were highly expressed in the flower and bud, while the unigenes grouped in Clusters 2 and 3 were highly expressed in the leaf and root of *A. carmichaelii*, respectively. It is noteworthy that Cluster 3, which showed high expression of grouped unigenes in the root, included unigenes annotated as AACT (AACT3, AACT4), HMGR (HMGR1, HMGR2, HMGR3), DXS (DXS1, DXS2, DXS4, DXS5), GGPPS (GGPPS1), CDPS (CDPS4), and KS (KS1, KS2), among which HMGR and DXS have been reported as the bottleneck enzymes of the MVA and MEP pathways, respectively [47]. Root-specific co-expression of CDPS and KS, key enzymes for the committed step towards biosynthesis of diterpene alkaloids, is consistent with previous reports on the accumulation of aconitine-type metabolites in the roots of *A. carmichaelii*. Several studies have reported that CDPS is involved in the tissue-specific accumulation of diterpene alkaloids [48,49]. Two CDPSs have been reported in rice to be localized differentially owing to their distinct biological role, with high expression in the vascular bundle tissues for GA biosynthesis (OsCPS1) and epidermal cells for the biosynthesis of phytoalexins (OsCPS2), respectively [50]. Since the root is the main tissue where we see the accumulation of aconitine-type diterpene alkaloids, our qualitative results suggesting high expression of unigenes annotated as the key enzymes of the aconitine-type diterpene alkaloids biosynthetic pathway seem to be quite significant. While unigenes corresponding to HMGR were also reported to be highly upregulated in roots of *A. heterophyllum* [18], the expression of GGPPS and CDPS were downregulated in the root compared to the shoot, and the expression of KS showed non-significant modulation in the two tissues [51]. These results indicate the probability of CDPS being the rate-limiting step towards the biosynthesis of aconitine-type diterpene alkaloids. In an attempt to elucidate the biosynthesis of atisine in *A. hetreophyllum*, Kumar et al. proposed that both kaurene and atisane undergo oxidation and subsequent hydroxylation to form steviol and atisenol, respectively. While kaurene oxidase (KO) and kaurenoic acid hydroxylase (KH) are characterized to catalyze the oxidation and hydroxylation reactions of kaurene, respectively, the enzyme responsible for catalyzing these reactions of atisane remains unknown. The incorporation of β-aminoethanol moiety in the atisane and steviol facilitates the formation of an atisine skeleton. While the accumulation of steviol has been reported in *A. heterophyllum*, it has not been detected in *A. carmichaelii* [51]. Consistent with this, we were also not able to identify the unigene annotated as kaurenoic acid hydroxylase in our transcriptome dataset, indicating that atisine biosynthesis in *A. carmichaelii* most likely takes the atisenol route.

Expression analysis of unigenes demonstrated that, while all enzyme coding genes of the MVA and MEP pathway were not highly expressed in the root, enzymes associated with aconitine-type diterpene alkaloid biosynthesis, namely, GGPPS, CDPS, and KS, showed high expression in the root of *A. carmichaelii*. Biosynthesis of aconitine-type diterpene alkaloids is not completely understood, except that it is derived from a kaurene/atisane ring formed through the cyclization of GGPP mediated by CDPS and KS. High expression of unigenes annotated as homologs of GGPPS, CDPS, and KS in roots, which accumulate diterpene alkaloids, suggests that roots are an active biosynthetic site. Our results led us to speculate that the precursors for aconitine-type diterpene alkaloids may be synthesized in tissues other than the root, which then are transported to the root, where they are acted upon by key enzymes including GGPPS, CDPS, and KS to form an atisine skeleton. Subsequently, further oxidations, as well as the loss of carbon atoms, may lead to a wide range of C_20_- and C_19_-type diterpene alkaloids. Several studies have previously reported the tissue compartmentalization of precursors, intermediates, and final products in highly specialized cells as well as in different plant tissues [52,53], and *A. carmichaelii* may have a similar strategy for synthesizing aconitine-type diterpene alkaloids. Further studies focusing on tracing the intermediates across different tissues of *A. carmichaelii* and functional characterization of enzymes involved in the biosynthesis of aconitine-type diterpene alkaloids would be required to establish whether the biosynthesis is localized to one tissue or a concerted interaction between different tissues is necessary.

### 2.9. Phylogenetic Analysis of Unigenes Annotated as Cytochrome P450 with Highest Expression in Root

**Figure 9:**
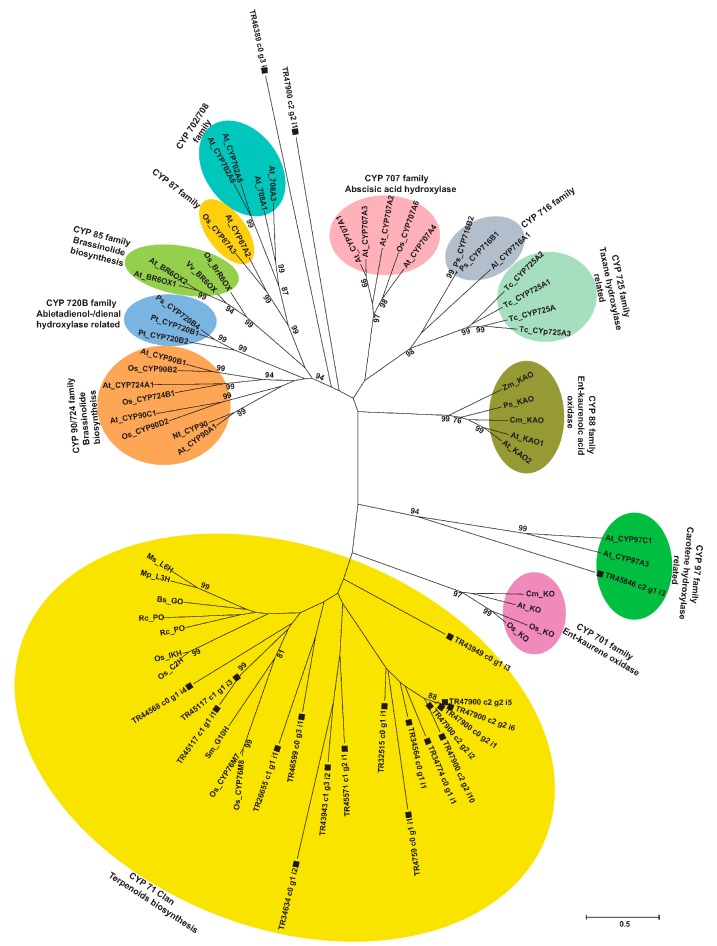
Phylogenetic analysis of unigenes, highly correlated with CDPS4 and KS2 expression and annotated as CYP450s, together with functionally characterized CYPs involved in the terpenoid biosynthesis and metabolism. The nucleotide sequences for unigenes annotated as CYP450s were translated and the corresponding protein sequences were aligned with 54 well-known CYPs using the MUSCLE program. The evolutionary history was inferred by the Maximum Likelihood method, based on the JTT matrix-based model with bootstrap values obtained after applying 1000 replications using the MEGA6 program. Bootstrap values above 75% are shown here. Initial tree(s) for the heuristic search were obtained by applying the Neighbor-Joining method to a matrix of pairwise distances estimated using a JTT model. The tree is drawn to scale, with branch lengths measured in the number of substitutions per site. The scale bar represents 0.5 estimated nucleotide changes per sequence position. Abbreviations: At, *Arabidopsis thaliana*; Os, *Oryza sativa*; Ps, *Pices sitchensis*; Pt, *Pinus taeda*; Vv, *Vitis vinifera*; Ps, *Pisum sativum*; Tc, *Taxus cuspidate*; Cm, *Cucurbita maxima*; Zm, *Zea mays*; Nt, *Nicotiana tabacum*; Al; *Arabidopsis lyrata*; Rc, *Ricinus communis*; Ms, *Mentha spicata*; Mp, *Mentha piperita*; Bs, *Barnadesia spinose*.

Terpenoid biosynthesis involves two major enzyme families, the terpene synthases and cytochrome P450s. Terpene synthase catalyzes cyclization of GGPP, which later is oxidatively functionalized by CYP450s through biochemical reactions including hydroxylation, alkylation, and esterification. CYP450s, therefore, play a decisive role in driving the diversification of the terpenoids with a wide range of bioactivity [54]. Although several studies in the past have identified and functionally characterized CYP450s involved in the biosynthesis of terpenoids such as taxane, tanshiones, and artemisinin, little is known about the different CYP450s involved in the biosynthesis of aconitine-type diterpene alkaloids. The annotated *A. carmichaelii* de novo transcriptome assembly included 124 unigenes annotated as cytochrome P450 with sequence length over 500 bps, alignment length over 100 bps, and sequence similarity with its corresponding homologs over 75%. Among these, 21 unigenes were highly correlated with CDPS4 and KS2 with a correlation coefficient over 0.85 and were highly expressed in roots (Table S12). Since the root of *A. carmichaelii* is the main tissue for the accumulation of aconitine-type diterpene alkaloids, for these unigenes with the highest expression in roots and co-expressed with CDPS4 and KS2, terpene synthase, which provides a substrate for enzymes belonging to the CYP450 family, represents a potential candidate for participating in its biosynthesis. Phylogenetic analysis of these 21 unigenes was performed together with 54 CYPs, which are known to participate in terpenoid biosynthesis in different plant species (Table S13). As a result, 18 of these unigenes were grouped as A type (CYP 71 clan), whereas three of them were classified as non-A type (Figure 9). Among the unigenes belonging to non-A type, TR45846|c2_g1_i3 was clustered in the CYP 97 family together with CYPs of *A. thaliana* participating in the carotene hydroxylation reaction, while two of them, TR47900|c2_g2_i1 and TR46389_c0_g3_i1, stood independently. Among various clans of cytochrome P450, the CYP71 clan is the largest clan in plants and contains more than 50% of all the cytochrome P450s identified so far. The majority of these are not yet functionally known, but those that have been characterized mainly participate as monoterpenoids, sesquiterpenoids, and diterpene alkaloids modifying enzymes [55,56]. Within the unigenes of *A. carmichaelii* that were clustered in the CYP71 clan, while the majority of them formed a separate and independent cluster from the rest of the CYP450s, TR44568|co_g1_i4, TR45117|c1_g1_i3, and TR45117|c1_g1_i1 were remarkable because they were the nearest neighbors to CYP76M7 and CYP76M8 of rice, which are reported to be located within the gene cluster together with CDPS and KS and participate in the hydroxylation of ent-cassadiene to form phytocassanes [57]. Co-clustering of these three unigenes with the CYPs involved in secondary metabolite production in rice may suggest their potential role in catalyzing the subsequent conversion of atisane to form atisenol, leading to the biosynthesis of atisine in *A. carmichaelii*. Further investigations would be required to confirm the function of these unigenes in the biosynthesis of aconitine-type diterpene alkaloids.

## 3. Materials and Methods

### 3.1. Plant Materials

*A. carmichaelii* plants (plant material No. 0871-09TS in NIBIOHN) were grown in natural conditions at the Tsukuba Division, Research Center for Medicinal Plant Resources, National Institutes of Biomedical Innovation, Health and Nutrition (NIBIOHN, Tsukuba, Japan). All four tissue types, namely flower, bud, leaf, and root, were harvested on ice in the month of September 2014 and snap frozen using liquid nitrogen before storing at −80 °C until RNA extraction.

### 3.2. RNA Isolation and cDNA Library Preparation

Frozen tissues of *A. carmichaelii* were used for RNA extraction and cDNA library preparation. RNA extraction, RNA integrity analysis, mRNA sample preparation, fragmentation of isolated mRNA, and cDNA library preparation for Illumina sequencing were performed as described previously [40]. 

### 3.3. Illumina Sequencing

The cDNA library thus prepared for each tissue of *A. carmichaelii* was sequenced using an Illumina HiSeq™ 2000 sequencer (Illumina, Inc., San Diego, CA, USA), and paired-end reads were obtained with an average length of 101 bps. Preparation and shearing of mRNA, cDNA library preparation, and sequencing were performed at Kazusa DNA Research Institute, Chiba, Japan. The raw sequence reads for all four tissues of *A. carmichaelii*, their expression value, and the de novo transcriptome assembly used in this study have been deposited in NCBI’s Gene Expression Omnibus (GEO), and are available at GEO series accession number GSE106247 (https://www.ncbi.nlm.nih.gov/geo/query/acc.cgi?acc=GSE106247).

### 3.4. RNA-Seq Raw Reads Pre-Processing, De Novo Transcriptome Assembly, and Functional Classification of Unigenes

Raw sequencing reads thus generated were preprocessed through the Trimmomatic program to remove adaptor sequences, short reads, reads with ambiguous ‘N’ base >5%, and low-quality reads (phred score < 30). Paired-end processed reads, as well as unpaired high-quality reads that lost their corresponding sequence partner due to Trimmomatic filtering, for all four tissue types were combined to build a de novo transcriptome assembly of *A. carmichaelii*. Three popular assemblers, namely CLC Genomics Workbench 8.0.3 (https://www.qiagenbioinformatics.com/), SOAPdenovo-Trans [24], and Trinity [25], were used for generating transcriptome assemblies. The de novo transcriptome assembly resulting from the Trinity program showed the best assembly statistics and was further processed through the CD-HIT-EST program [26] for sequence redundancy removal, and subsequently used for annotation and characterization. The unigenes thus obtained were used for the read alignment and abundance estimation in individual tissues of *A. carmichaelii* using Bowtie 2.0 [38] and RSEM [39], respectively. Unigene expression was calculated in terms of Fragments per Kilobase exon per million mapped fragments (FPKM). Correlation analysis was performed for all four tissues through the DeSeq2 program of the R-package [58]. All heat maps depicting the expression levels of unigenes across the four tissue types were created using Heatmap2.0 of R-package.

The de novo transcriptome assembly was annotated using a Blastx-based homology search against the NCBI-nr database with an *E*-value cutoff <10^−5^, and the top Blastx hit was used to assign sequence description and putative functionality to the unigenes. Further, Blast2GO was used to obtain GO terms, EC number, and KEGG pathway-based annotation for the unigenes of *A. carmichaelii*. For the identification of unigenes involved in the aconitine-type diterpene alkaloid biosynthesis, the annotated transcriptome assembly was screened for the homologs of the associated enzymes, and candidate unigenes were selected that had a length greater than 500 bps and a sequence similarity with top Blastx hit of over 70%. 

### 3.5. Identification of Simple Sequence Repeats (SSRs)

The de novo transcriptome assembly of *A. carmichaelii* was scanned for simple sequence repeats using the Microsatellite Identification Tool (MISA) [59] with the search parameters described previously [40]. The SSRs for *A. heterophyllum* were also identified using the approach described above.

### 3.6. GO Enrichment Analysis 

Tissue-specific unigenes of *A. carmichaelii* with non-zero FPKM values as the test set were used against unigenes of *A. carmichaelii* with GO annotation as the reference set to perform GO enrichment analysis using Blast2GO [30] as described previously [40]. GO enrichment analysis based on Fisher’s exact test was performed with a *p*-value cutoff set at 0.05. Furthermore, a hypergeometric test with Benjamini and Hochberg false discovery rate correction was also performed. GO enrichment analysis of unigenes of *A. carmichaelii* shared between *A. carmichaelii* and *A. heterophyllum*, and of unigenes specific to *A. carmichaelii*, was performed using a similar approach to that described above.

### 3.7. KEGG Pathway Enrichment Analysis

For KEGG pathway enrichment analysis, all the unigenes of *A. carmichaelii* mapped on to the KEGG pathway and the tissue-specific unigenes mapped on to the pathway were selected as the reference set and test set, respectively. In order to identify the significantly enriched KEGG pathway, a hypergeometric test was used to calculate the p-value using the following formula:

$$P\;\left(X=x\right)=h\;\left(x;n,M,N\right)=\frac{\left(\begin{array}{c} M\\ x \end{array}\right)\;\left(\begin{array}{c} N-M\\ n-x \end{array}\right)}{\left(\begin{array}{c} N\\ n \end{array}\right)}.$$

Note: *x* represents the number of unigenes mapped to a certain KEGG pathway in the test set, *n* represents total number of unigenes in the test set mapped to KEGG pathway, *M* represents the number of unigenes in the reference set mapped to a certain KEGG pathway and *N* represents the total number of unigenes in a reference set mapped to the KEGG pathway.

### 3.8. Comparative Transcriptome Analysis of A. carmichaelii and A. heterophyllum 

The de novo transcriptome assemblies of *A. carmichaelii* and *A. heterophyllum* were translated into corresponding protein sequences by picking a translational frame that was used for annotation based on the Blastx results or resulting in longer amino acids. The translated proteins thus obtained were used to identify conserved orthogroups between the two plant species using OrthoFinder with its default parameters [41]. Orthologs and unique sequences were determined for both individual species as well as two-way comparisons.

### 3.9. Phylogenetic Analysis

The unigene of *A. carmichaelii* denoted as cytochrome P450, having a sequence length of over 500 bps, alignment length and sequence similarity with the top Blastx hit of over 100 bps and 75%, respectively, and high expression correlation with CDPS4 and KS2, was selected for phylogenetic analysis. These selected unigenes were translated to their corresponding protein sequences by selecting the translation frame that resulted in the longest amino acid sequence while starting with methionine using Biology Workbench online tool (http://workbench.sdsc.edu/). Protein sequences of cytochrome P450 annotated unigenes and previously characterized cyp450s involved in the biosynthesis of terpenoids in other plant species were aligned using the MUSCLE program, and evolutionary distances were inferred using the maximum likelihood method, based on a Jones–Taylor–Thornton (JTT) matrix-based model with bootstrap values obtained after 1000 replications using MEGA6 software [60].

## 4. Conclusions

In this study, we established de novo transcriptome assembly of *A. carmichaelii* using four tissue types, namely flower, bud, leaf, and root. Through sequence-homology-based transcriptome annotation coupled with co-expression analysis, we identified potential candidate unigenes involved in the biosynthesis of aconitine-type diterpenoids. Transcript abundance analysis showed unigenes corresponding to the key enzymes, catalyzing the committed step in the biosynthesis of diterpenoids, to be highly expressed in the root. We also identified candidate unigenes, designated as cytochrome P450s, which putatively are involved in the diversification of aconitine-type metabolites. Moreover, comparative transcriptomics analysis using de novo transcriptome assemblies of *A. carmichaelii* with *A. heterophyllum* enabled us to identify orthologue genes between these two plant species. Interestingly, GO enrichment analysis of the orthologue genes revealed essential biological processes together with secondary metabolic processes to be highly enriched. In this first deep transcriptome analysis of aconitine producing medicinal plant *A. carmichaelii*, we report potential candidate genes associated with aconitine and aconitine-derived diterpene alkaloids highly expressed in the roots. This study, being the first deep transcriptomic resource of *A. carmichaelii*, lays the foundation for future studies on the functional characterization of candidate genes involved in the biosynthesis of toxic aconitine-type diester–diterpenoids, which could improve the production of pharmaceutically important metabolites through metabolic engineering.

## Figures and Tables

**Table 1:** Transcriptome assembly statistics for *A. carmichaelii*.

Description	Transcripts
Number of transcripts	128,183
Total assembled bases	78,623,549
Average length (bps)	613.37
Median length (bps)	383
Maximum length (bps)	13,485
Minimum length (bps)	224
N50 (bps)	830
GC content	42.35

**Table 2:** SSRs detected in the transcriptome assembly of *A. carmichaelii*.

Results of SSR Searches	
Total number of sequences examined	128,183
Total number of identified SSRs	16,068
Number of SSR containing sequences	14,168
Number of sequences containing more than one SSR	1675
Number of SSRs present in the compound formation	686

**Table 3:** Comparative analysis of transcriptome of *A. carmichaelii* with that of *A. heterophyllum* through OrthoFinder. The nucleotide sequences for both plants were translated and the corresponding protein sequences were obtained, using the longest frame, starting with methionine, and were further used for OrthoFinder analysis.

Total number of orthogroups: 20,247			
Mean orthogroup size: 2.8			
Median orthogroup size: 2.0			
**Description**	**Overall**	***A. heterophyllum***	***A. carmichaelii***
Number of genes	209,790	81,607	128,183
Number of genes in orthogroups	56,915	26,282	30,633
Number of unassigned genes	152,875	55,325	97,550
Percentage of genes in orthogroups	27.1	32.2	23.9
Percentage of unassigned genes	72.9	67.8	76.1
Number of orthogroups containing species	20,232	20,239	20,240
Number of species-specific orthogroups	15	8	7
Number of genes in species-specific orthogroups	87	39	48

**Table 4:** Summary of unigenes of *A. carmichaelii* involved in the biosynthesis of aconitine-type diterpene alkaloids.

Enzymes	Enzyme Name	Unigene	Unigene Length	Similarity
**Mevalonate Pathway (MVA Pathway)**				
AACT1	Acetyl-CoA acetyltransferase	TR41842|c0_g3_i5	950	96.21212
AACT2		TR41842|c0_g3_i3	957	93.61702
AACT3		TR44607|c2_g6_i4	962	94.29658
AACT4		TR44607|c2_g6_i2	1057	94.27481
HMGS1	Hydroxymethylglutaryl-CoA synthase	TR19454|c0_g1_i1	571	85.81081
HMGS2		TR40295|c3_g2_i3	909	87.21805
HMGS3		TR40295|c3_g2_i1	1761	91.45299
HMGS4		TR40318|c0_g1_i1	1992	84.52656
HMGR1	Hydroxymethylglutaryl-CoA reductase	TR44439|c4_g1_i5	784	72.42647
HMGR2		TR44439|c4_g1_i7	2118	89.20354
HMGR3		TR44439|c4_g1_i1	2179	88.64028
MVK1	Mevalonate kinase	TR45020|c0_g1_i1	1335	86.23377
PMK1	Phosphomevalonate kinase	TR45726|c0_g1_i3	575	83.50515
PMK2		TR45726|c0_g1_i1	641	83.50515
PMK3		TR45726|c0_g1_i12	2237	82.47012
MVDD1	Mevalonate diphosphate decarboxylase	TR44355|c0_g1_i1	1781	91.23223
MVDD2		TR44355|c0_g1_i3	1797	91.46919
**Methylerythritol Pathway (MEP Pathway)**				
DXS1	1-deoxy-d-xylulose-5-phosphate synthase	TR28848|c0_g1_i1	505	95.83333
DXS2		TR32192|c0_g1_i2	532	86.36364
DXS3		TR47591|c0_g1_i2	554	77.77778
DXS4		TR15579|c0_g1_i1	563	97.3262
DXS5		TR32608|c0_g1_i1	578	96.33508
DXS6		TR47591|c0_g1_i5	648	77.77778
DXS7		TR29244|c0_g2_i2	668	78.18182
DXS8		TR47591|c0_g1_i3	866	77.77778
DXS9		TR47591|c0_g2_i1	2330	95.54235
DXS10		TR43248|c0_g1_i2	2462	94.00279
DXS11		TR42479|c0_g1_i1	2585	84.34903
DXR1	1-deoxy-d-xylulose-5-phosphate reductoisomerase	TR38806|c0_g1_i1	2065	92.11087
ISPD1	2-*C*-methyl-d-erythritol 4-phosphate cytidylyltransferase	TR34080|c0_g1_i3	1173	95.54455
ISPD2		TR34080|c0_g1_i1	1229	95.04505
ISPD3		TR34080|c0_g1_i6	1355	95.47511
ISPE1	4-(cytidine-5′-diphospho)-2-*C*-methyl-d-erythritol kinase	TR43665|c0_g1_i2	1549	84.79381
ISPF1	2-*C*-methyl-d-erythritol 2,4-cyclodiphosphate synthase	TR34396|c0_g1_i1	945	90.37433
ISPG	(*E*)-4-hydroxy-3-methylbut-2-enyl diphosphate synthase	TR42806|c0_g1_i1	2806	94.22043
ISPH1	(*E*)-4-hydroxy-3-methylbut-2-enyl diphosphate reductase	TR42285|c1_g1_i2	1718	91.16379
IPPI1	Isopentenyl diphosphate isomerase	TR37870|c0_g1_i1	1326	87.33333
IPPI2		TR44603|c0_g1_i3	2692	88.98848
**Aconitine-Type Diterpene Alkaloid Biosynthetic Pathway**				
GGPPS1	Geranylgeranyl pyrophosphate synthase	TR47410|c1_g2_i2	1384	83.58663
GGPPS2		TR35223|c0_g1_i1	1172	88.60759
GGPPS3		TR44055|c0_g1_i3	765	92.24806
GGPPS4		TR44055|c0_g1_i1	1496	89.59732
GGPPS5		TR46755|c1_g1_i1	1752	88.33819
GGPPS6		TR42099|c0_g1_i1	1532	81.73077
GGPPS7		TR46755|c0_g1_i3	592	75.71429
CDPS1	ent-copalyl diphosphate synthase	TR48303|c1_g1_i4	748	71.42857
CDPS2		TR48303|c0_g1_i1	752	80.32787
CDPS3		TR48303|c1_g1_i3	1512	79.66903
CDPS4		TR36243|c0_g1_i1	2342	73.64238
KS1	Kaurene synthase	TR44693|c0_g3_i1	1120	72.12121
KS2		TR44693|c0_g1_i2	1539	75.80645
KS3		TR48214|c4_g1_i1	2144	77.95031

## Supplementary Materials

Supplementary Materials are available online.
