# Supplementary material for: De Novo RNA Sequencing and Expression Analysis of Aconitum carmichaelii to Analyze Key Genes Involved in the Biosynthesis of Diterpene Alkaloids
Source: Molecules. 2017 Dec 5;22(12):2155. doi: 10.3390/molecules22122155 (PMC6150021; doi:10.3390/molecules22122155)
Supplement: Supplementary file 1 [file molecules-22-02155-s001.zip › supplementary-revised/ESM_13_v1.pdf]

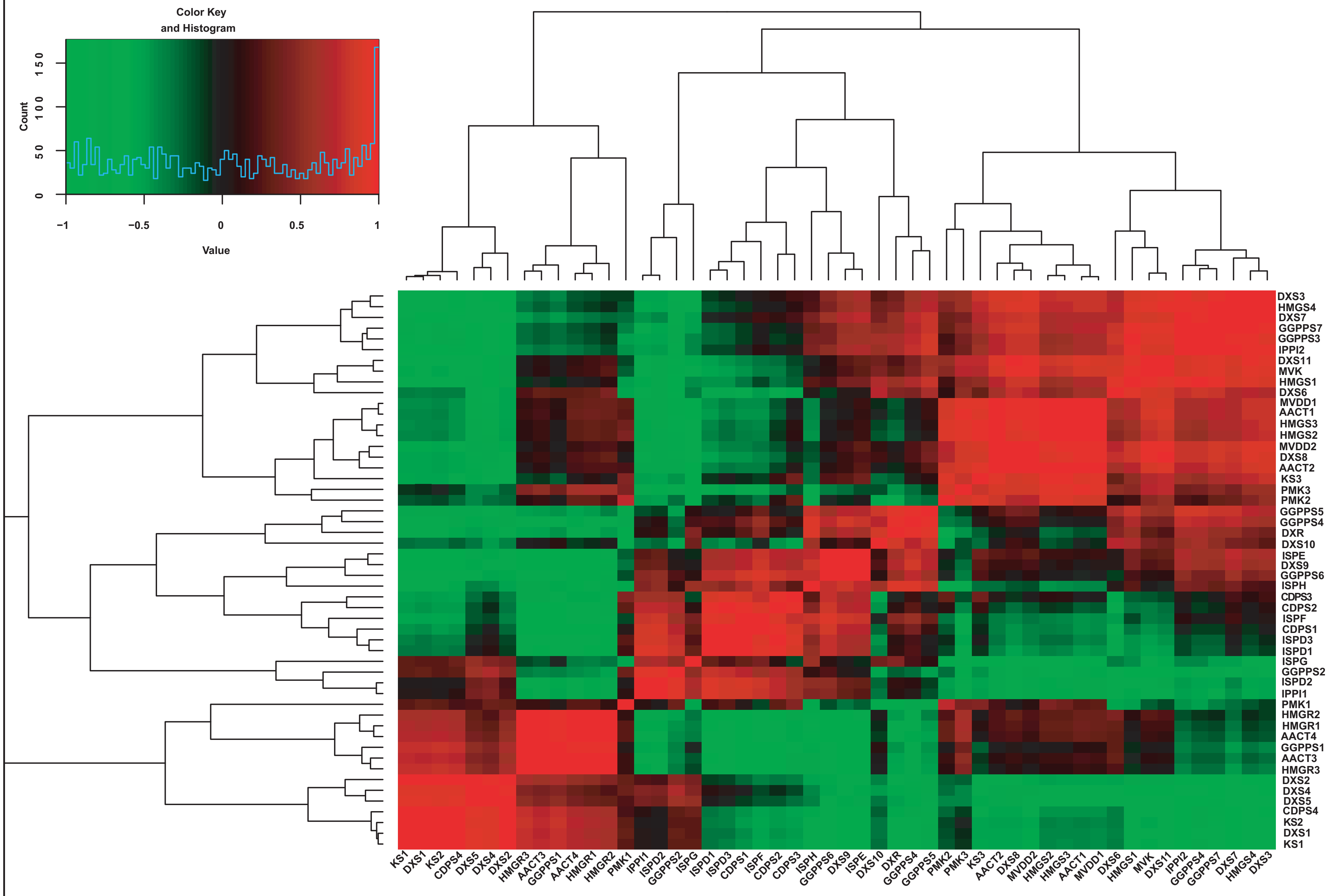

Color Key
and Histogram
Count
0
50
100
150
−1
−0.5
0
0.5
1
Value
DXS3
HMGS4
DXS7
GGPPS7
GGPPS3
IPPI2
DXS11
MVK
HMGS1
DXS6
MVDD1
AACT1
HMGS3
HMGS2
MVDD2
DXS8
AACT2
KS3
PMK3
PMK2
GGPPS5
GGPPS4
DXR
DXS10
ISPE
DXS9
GGPPS6
ISPH
CDPS3
CDPS2
ISPF
CDPS1
ISPD3
ISPD1
ISPG
GGPPS2
ISPD2
IPPI1
PMK1
HMGR2
HMGR1
AACT4
GGPPS1
AACT3
HMGR3
DXS2
DXS4
DXS5
CDPS4
KS2
DXS1
KS1
KS1
DXS1
KS2
CDPS4
DXS5
DXS4
DXS2
HMGR3
AACT3
GGPPS1
AACT4
HMGR1
HMGR2
PMK1
IPPI1
ISPD2
GGPPS2
ISPG
ISPD1
ISPD3
CDPS1
ISPF
CDPS2
CDPS3
ISPH
GGPPS6
DXS9
ISPE
DXS10
DXR
GGPPS4
GGPPS5
PMK2
PMK3
KS3
AACT2
DXS8
MVDD2
HMGS2
HMGS3
AACT1
MVDD1
DXS6
HMGS1
MVK
DXS11
IPPI2
GGPPS4
GGPPS7
DXS7
HMGS4
DXS3
