# Supplementary material for: De Novo RNA Sequencing and Expression Analysis of Aconitum carmichaelii to Analyze Key Genes Involved in the Biosynthesis of Diterpene Alkaloids
Source: Molecules. 2017 Dec 5;22(12):2155. doi: 10.3390/molecules22122155 (PMC6150021; doi:10.3390/molecules22122155)
Supplement: Supplementary file 1 [file molecules-22-02155-s001.zip › supplementary-revised/ESM_1_v1.pdf]

Table S1 - Summary of Trimmomatic output for *Aconitum carmichaelii*

Description	Flower	Bud	Leaf	Root
**Input Read Pairs**	9,294,842	8,953,957	6,816,584	6,125,840
**Both Surviving**	8,693,350 (93.53%)	8,229,917 (91.91%)	6,432,224 (94.3%)	5,671,296 (92.58%)
**Forward Only Surviving**	523,573 (5.63%)	644,435 (7.2%)	325,541 (4.78%)	405,374 (6.62%)
**Reverse Only Surviving**	47,159 (0.57%)	38,298 (0.43%)	33,314 (0.49%)	27,027 (0.44%)
**Dropped**	30,760 (0.33 %)	41,307 (0.46%)	25,505 (0.37%)	22,143 (0.36%)
