# Supplementary material for: De Novo RNA Sequencing and Expression Analysis of Aconitum carmichaelii to Analyze Key Genes Involved in the Biosynthesis of Diterpene Alkaloids
Source: Molecules. 2017 Dec 5;22(12):2155. doi: 10.3390/molecules22122155 (PMC6150021; doi:10.3390/molecules22122155)
Supplement: Supplementary file 1 [file molecules-22-02155-s001.zip › supplementary-revised/ESM_2_v1.pdf]

Table S2: Summary of *de novo* transcriptome assembly statistics of *Aconitum carmichaelii* resulting from the three different assemblers

Assembler	Kmer	No. of contigs	N50	Min length	Max length	Total
Trintiy	25	182,928	905	224	13485	117.4e6
CLC-work bench	25	88,873	554	200	9463	44.1e6
SOAPdenovo-	31	268,504	682	200	10527	39.53e6
Trans	41	255,462	605	200	10099	40.44e6
	51	253,918	538	200	9372	40.35e6
	63	171,233	466	200	9421	38.39e6
	71	117,985	454	200	9497	32.52e6
	91	11,696	489	200	9450	4.65e6
