# Supplementary material for: De Novo RNA Sequencing and Expression Analysis of Aconitum carmichaelii to Analyze Key Genes Involved in the Biosynthesis of Diterpene Alkaloids
Source: Molecules. 2017 Dec 5;22(12):2155. doi: 10.3390/molecules22122155 (PMC6150021; doi:10.3390/molecules22122155)
Supplement: Supplementary file 1 [file molecules-22-02155-s001.zip › supplementary-revised/ESM_7_v1.pdf]

Table S7: Statistics of SSRs detected in the transcriptome assembly of *A. heterophyllum*

**Results of SSR searches**	
Total number of sequences examined	81607
Total number of identified SSRs	9068
Number of SSR containing sequences	8026
Number of sequences containing more than 1 SSR	888
Number of SSRs present in compound formation	494

**Distribution of different repeat type classes**	
Mono-nucleotides	5713
Di-nucleotides	985
Tri-nucleotides	2187
Tetra-nucleotides	58
Penta-nucleotides	28
Hexa-nucleotides	97
