# Supplementary figures and images for: De Novo RNA Sequencing and Expression Analysis of Aconitum carmichaelii to Analyze Key Genes Involved in the Biosynthesis of Diterpene Alkaloids
Source: Molecules. 2017 Dec 5;22(12):2155. doi: 10.3390/molecules22122155 (PMC6150021; doi:10.3390/molecules22122155)

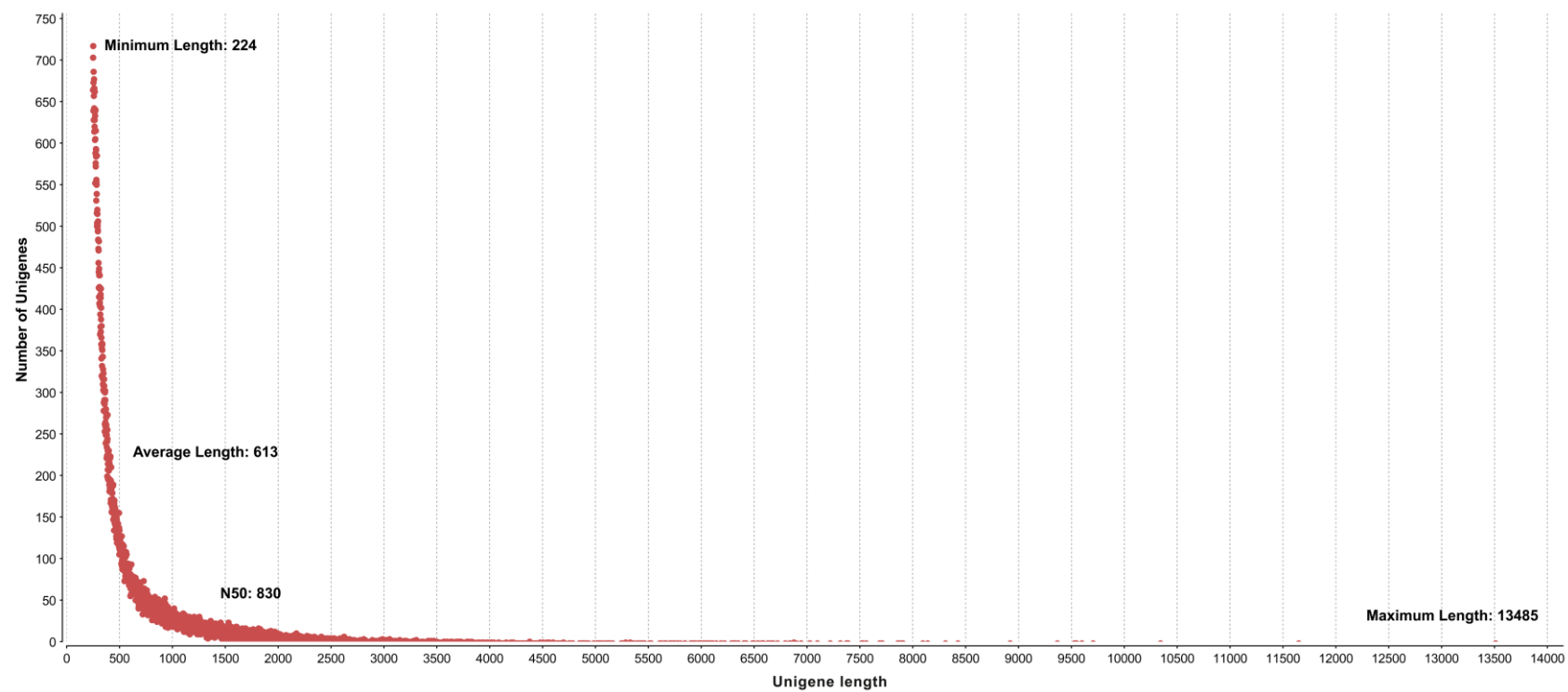

Supplement: Supplementary file 1 [file molecules-22-02155-s001.zip › supplementary-revised/ESM_3_v1.pdf]
